# Machine learning models identify ferroptosis-related genes as potential diagnostic biomarkers for Alzheimer’s disease

**DOI:** 10.3389/fnagi.2022.994130

**Published:** 2022-09-28

**Authors:** Yanyao Deng, Yanjin Feng, Zhicheng Lv, Jinli He, Xun Chen, Chen Wang, Mingyang Yuan, Ting Xu, Wenzhe Gao, Dongjie Chen, Hongwei Zhu, Deren Hou

**Affiliations:** ^1^Department of Rehabilitation, The First Hospital of Changsha, Changsha, China; ^2^Department of Neurology, The Third Xiangya Hospital, Central South University, Changsha, China; ^3^Department of Neurosurgery, The First People’s Hospital of Chenzhou, Chenzhou, China; ^4^Department of Hepatopancreatobiliary Surgery, The Third Xiangya Hospital, Central South University, Changsha, China

**Keywords:** Alzheimer’s disease, ferroptosis, diagnostic model, bioinformatics, machine learning algorithms

## Abstract

Alzheimer’s disease (AD) is a complex, and multifactorial neurodegenerative disease. Previous studies have revealed that oxidative stress, synaptic toxicity, autophagy, and neuroinflammation play crucial roles in the progress of AD, however, its pathogenesis is still unclear. Recent researches have indicated that ferroptosis, an iron-dependent programmed cell death, might be involved in the pathogenesis of AD. Therefore, we aim to screen correlative ferroptosis-related genes (FRGs) in the progress of AD to clarify insights into the diagnostic value. Interestingly, we identified eight FRGs were significantly differentially expressed in AD patients. 10,044 differentially expressed genes (DEGs) were finally identified by differential expression analysis. The following step was investigating the function of DEGs using gene set enrichment analysis (GSEA). Weight gene correlation analysis was performed to explore ten modules and 104 hub genes. Subsequently, based on machine learning algorithms, we constructed diagnostic classifiers to select characteristic genes. Through the multivariable logistic regression analysis, five features (RAF1, NFKBIA, MOV10L1, IQGAP1, FOXO1) were then validated, which composed a diagnostic model of AD. Thus, our findings not only developed genetic diagnostics strategy, but set a direction for further study of the disease pathogenesis and therapy targets.

## Introduction

Alzheimer’s disease (AD), a progressive neurodegenerative disorder, is the most major form of dementia. Clinically, AD is characterized by cognitive impairments, language deficits and behavioral disturbances (Villain and Dubois, 2019). As per Alzheimer’s Disease International in 2019, approximately 50 million people suffer from AD. Thus, the therapeutic methods of AD need to be explored urgently. For decades, most mechanism explanations have focused on amyloid-β accumulation and neurofibrillary tangles. However, medicine effect of inhibiting amyloid plaque formation is less effective. Recent theoretical developments have revealed that oxidative stress (Chen and Zhong, 2014) also play a significant part in AD, in addition to synaptic toxicity (Hampel et al., 2018), autophagy (Zhang et al., 2021), neuroinflammation (Calsolaro and Edison, 2016). Almost all neurodegenerative diseases are associated with reactive oxygen species (ROS) (Patten et al., 2010).

Ferroptosis is defined as a form of programmed cell death driven by lipid peroxidation and this term first appeared in 2012 (Dixon et al., 2012). Since then, the field of ferroptosis has met with great discoveries in molecular mechanisms. Ferroptosis highly depends on two main physiological processes, i.e., cell metabolism (especially lipids, iron, and amino acids) and degradation (especially autophagy and the ubiquitin-proteasome system) (Chen et al., 2021). Moreover, exhaustion of glutathione and activity reduction of glutathione peroxidase 4 (GPX4) are crucial regulators in the occurrence of ferroptosis (Yang et al., 2014). It is worth noting that GPX4 can remove lipid peroxides (Ursini et al., 1982). Therefore, as GPX4 function is inhibited, ROS accumulates and promotes cell death.

Researchers have clarified that ferroptosis might participate in multiple diseases, such as cancer, neurodegeneration and ischemia/reperfusion (Yan et al., 2021). In recent years, it has attracted enormous interests in the relationship between ferroptosis and AD. Some studies have stated there is down-regulated expression of ferroportin1, excessive iron accumulation, and ROS generation in the AD mice and AD patients (Zhang et al., 2012; Bao et al., 2021; Majernikova et al., 2021), which suggest ferroptosis might be interrelated in the etiology of AD. The relationship between pathogenesis of ferroptosis and AD was revealed as the evidence of iron dyshomeostasis, enhanced lipid peroxidation and an impaired glutamate system (Long et al., 2022). Up till now, it remains challenges for pathological hypotheses of AD, therefore the regulation mechanisms of ferroptosis need draw more attention and further study. Genetic data could yield new insights into AD. However, expression patterns of controlling genes remain unclear, which limits further study of different biological processes. The issue that genetic screening is a diagnostic method or not still requires investigation.

In this study, we used Gene Expression Omnibus (GEO) and the Molecular Signatures Database (MsigDB) to identify the expression of ferroptosis-related genes (FRGs). Then, we investigated the co-expression network, performing weighted gene co-expression network analysis (WGCNA), Gene Ontology (GO) and Kyoto Encyclopedia of Genes and Genomes (KEGG) functional analysis. Hub genes associated with AD was explored and investigated the potential biological functions. To assess their influence on the diagnostic module, we constructed diagnostic classifiers based on multiple machine learning algorithms to perform feature selection and evaluated the diagnostic value of AD predictive model by multivariable logistic regression analysis. The major contribution of our work was the discovery of 5 FRGs which may be involved in the diagnosis of AD.

## Materials and methods

### Data set collection and differential expression analysis

With the GEOquery package, the RNA sequencing dataset (GSE33000) containing 310 samples was downloaded from the GEO database.^1^ Transcriptome data of GSE5281 and GSE48350 was extracted for confirmatory studies. GEO database is the largest and most comprehensive public gene expression database, which are freely available. We downloaded the genes of ferroptosis-related pathways from the MSigDB and identified a total of 60 genes related to ferroptosis^2^ (Liberzon et al., 2015). Gene expression profiles obtained from datasets were analyzed to seek DEGs with the limma R package. | log2FC| > 0.1 and *p*-value >0.05 as the cut−off criterion were applied to screen DEGs. Expressions of DEGs were visualized in Volcano plots and heatmaps.

### Consensus clustering

Consensus clustering is a useful method to discover biological characteristics in bioinformatics analysis. We selected FRGs for further analysis of different molecular subgroups in AD. Based on expressed FRGs selected above, a consistency matrix was built to identify the ferroptosis-related subtypes. The ConsensusClusterPlus package was used to divide the samples into diverse clusters with number set to 2. Cumulative distribution function (CDF) and area under CDF curve were used to select the optimal cluster number.

### Gene set enrichment analysis

Gene set enrichment analysis (GSEA)^3^ was employed to identify different functional phenotypes, which was performed using the “ClusterProfiler” R package (Wu et al., 2021). The reference genes performed GSEA are AD vs Normal and Cluster1 vs Cluster2. Additionally, the nominal (NOM) *p*-value <0.05 was considered to be significant.

### Weighted gene co-expression network analysis

WGCNA was applied to construct the expression patterns of genes from samples. A co-expressed gene module was composed of similar expression patterns with R-Studio software (Langfelder and Horvath, 2008). The R function “pickSoftThreshold” algorithm was utilized to select an appropriate soft threshold (β) modules were identified with hierarchical clustering and dynamic tree cut function. Gene significance (GS) and Module Membership (MM) derived from module eigengenes were defined as index of selecting hub genes.

### Functional and pathway enrichment analysis

GO and KEGG enrichment analyses were performed on the module most relevant to the AD, using the“clusterProfiler” and “enrichplot” on R Studio. Significance was adjusted to *P*-value <0.05.

### Construction and validation of classifier model

Patients from GSE33000 were first randomly grouped into train (70%) and test (30%) datasets by R package “caret” and function createFolds. GSE48350 and GSE5281 were chosen as external validation dataset. LASSO regression, random forest, XGBoost and Support Vector Machines (SVM) were performed for feature selection to build the diagnostic model using the most representative genes. R packages including “glmnet,” “Boruta,” “xgboost” as well as “e1071” were applied for this study.

After taking the intersection of the four machine learning algorithms, the remaining features were applied to construct an AD diagnostic model through Logistic regression. Diagnostic scores are calculated based on the following formula:


$$\mathrm{Diagnositc}\,\mathrm{Model}=\sum_{i=1-5}^{E\,x\,p_{i}}E\,x\,p_{i}\times c\,o\,e\,f_{i}$$

*i* the number of diagnostic genes; Exp standardized gene expression; coef regression coefficients.

The receiver operating curve (ROC) curve of the predicted results is drawn using the “pROC” R package. By calculating the area under the ROC curve (AUC), we determine the classification capability of the diagnostic model.

## Results

### Expression analysis and clustering of ferroptosis-related genes

**FIGURE 1:**
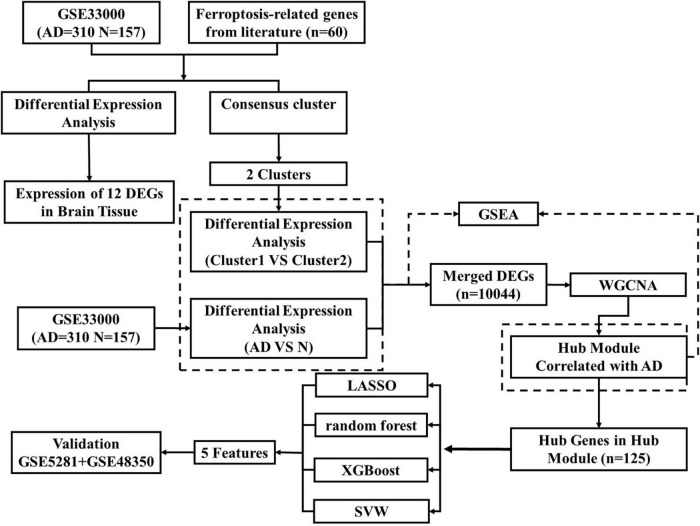
The overview of the whole study.

**FIGURE 2:**
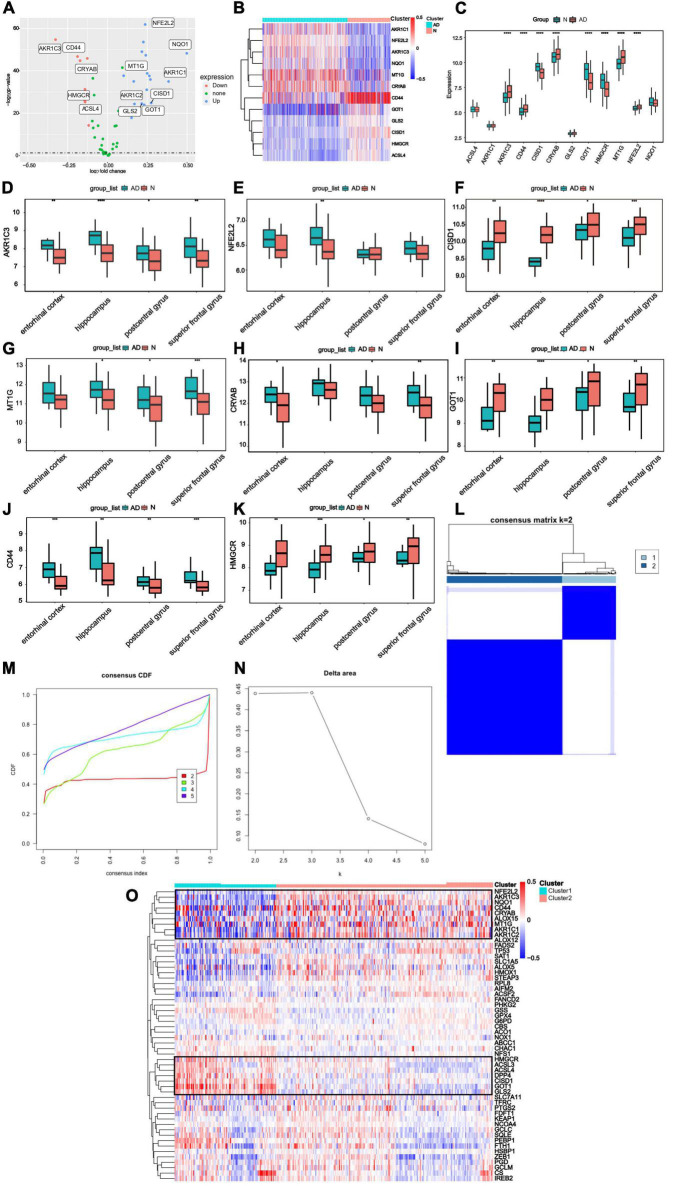
Differential expressing analysis and consensus clustering analysis of ferroptosis regulating genes **(A)** DEGs were demonstrated in the volcano plot. Up-regulated genes were represented in blue and down-regulated genes were represented in red. The markers of the most different expressing genes were labeled in this diagram. **(B)** The expression profiles for 12 labeled genes. Red represented the increasing expression and blue represented decreasing expression. **(C)** The expression levels of 12 labeled genes were demonstrated with boxplots in GSE5281 and GSE48350, and it showed that eight FRGs had significant expression levels compared to the AD and Normal tissues. (Median line evaluated expression level and * indicted significant difference). **(D–K)** The expression level of genes was analyzed in entorhinal cortex, hippocampus, postcentral gyrus and superior frontal gyrus. **(L)** Consistent clustering at the index *k* = 2. **(M)** The cumulative distribution function (CDF) of clustering (*k* = 2–5). **(N)** Delta area plot depicting the relative change under the CDF curve (*k* = 2–5). **(O)** The expression level of FRGs in different clusters (The left side was cluster1, while the right side was cluster2).

The workflow of this study was shown in (Figure 1). Based on GSE33000, Differential expression analysis was carried out to describe genetic differences in FRGs between AD and control samples. As can be seen from the volcano plot (Figure 2A), we discovered differentially expressed genesamong 60 FRGs. 13 differentially expressed genes (DEGs) were identified, of which 8 genes were upregulated and five genes were downregulated (*p* < 0.05, abs (logFC) > 0.1). The collection of 12 genes in 60 FRGs were listed in Figure 2B. The expression level of 12 labeled genes in AD and normal samples was verified by a boxplot. According to statistics mentioned above, eight FRGs were all significantly differently expressed between AD group and normal samples, suggesting the eight genes may play a vital role in the progress of AD regulated by ferroptosis (Figure 2C). Among the eight DEGs, the overexpression of genes (AKR1C3, CD44, CRYAB, MT1G, NFE2L2) was observed in AD tissues compared to normal tissues, and the rest of genes (CISD1, GOT1, HMGCR) was lowly expressed in AD. Besides, we also identified the eight significant genes expressed differently in several regions of brain tissue (Figures 2D–K).

Furthermore, to illustrate distinct expression patterns of FRGs among different AD patients, a consensus clustering was performed based on 60 FRGs and GSE33000 dataset. *k* = 2 was determined to have the best stability and reliability (Figures 2L–N). Gene expression profile was classified into two subtypes, including cluster1 and cluster2 (*n*1 = 99, *n*2 = 211). The heatmap indicated that expression of 60 FRGs among two clusters was represented (Figure 2O).

### Identification of ferroptosis-related genes-related differentially expressed genes

**FIGURE 3:**
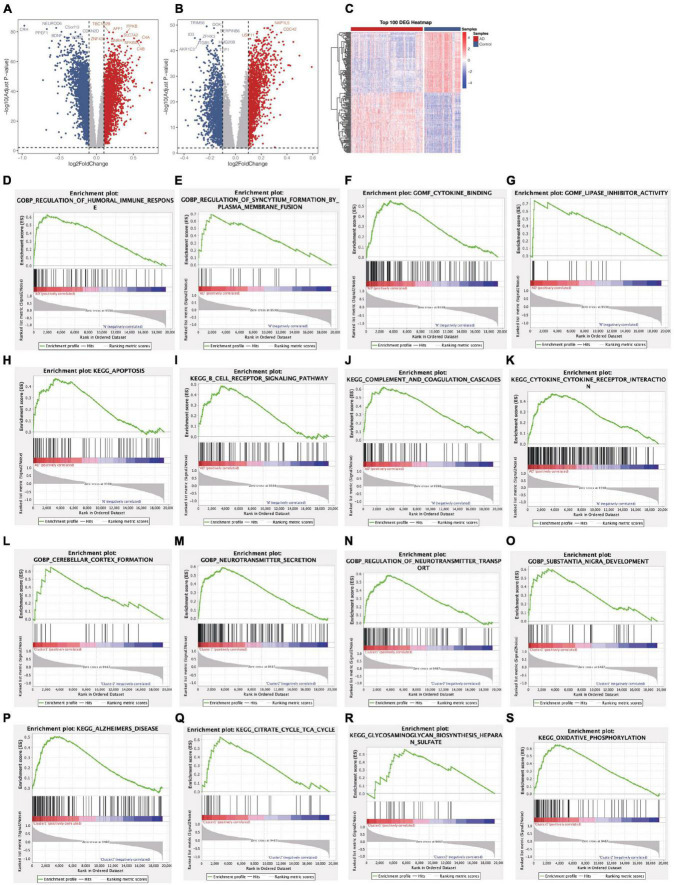
Identification of DEGs and Gene Set Enrichment Analysis from two groups. **(A)** Volcano plot of DEGs between AD patients and control samples (AD vs Normal). **(B)** Volcano plot of DEGs between Cluster1 and Cluster2 (Cluster1 vs Cluster2). **(C)** A heatmap of the top 100 DEGs. **(D–K)** Biological functions and pathways of genes between AD and normal samples. **(L–S)** Biological functions and pathways of genes between cluster1 and cluster2.

Through differential expression analysis of GSE33000, multiple genes were significantly differentially expressed in 310 AD patients compared with 157 control samples. DEGs were identified with thresholds of |log2FC| > 0.1 and *p*-value <0.05. As demonstrated in (Figure 3A), the DEGs consisted of 6,198 up-regulated genes and 7,050 down-regulated genes. Furthermore, compared to Cluster1, 11,505 DEGs were observed in Cluster2, containing 5,727 up-regulated genes and 5,778 down-regulated genes (Figure 3B). We set the intersection of the two groups of differential genes and a heatmap depicted the top 100 DEGs at the intersection between the two groups (Figure 3C).

### Gene set enrichment analysis identifies biological functions and pathways

Subsequently, we conduct GSEA analysis to investigate the significantly differential functions and pathways of the two groups. To explore the different functions and pathways in the AD, the KEGG pathways suggested that apoptosis, B cell receptor signaling pathway, complement and coagulation cascades, cytokine-cytokine receptor interaction were significantly enriched. The enriched GO terms included regulation of humoral immune response, regulation of syncytium formation by plasma membrane fusion, cytokine binding, lipase inhibitor activity (Figures 3D–K). In the enrichment of GO and KEGG collection between two clusters, the results revealed the pathways of Alzheimer’s disease, citrate cycle, TCA cycle, glycosaminoglycan biosynthesis heparan sulfate, oxidative phosphorylation. Further cerebellar cortex formation, neurotransmitter secretion, neurotransmitter transport, substantia nigra development were significantly enriched (Figures 3L–S).

### Construction of co-expression network and related modules

**FIGURE 4:**
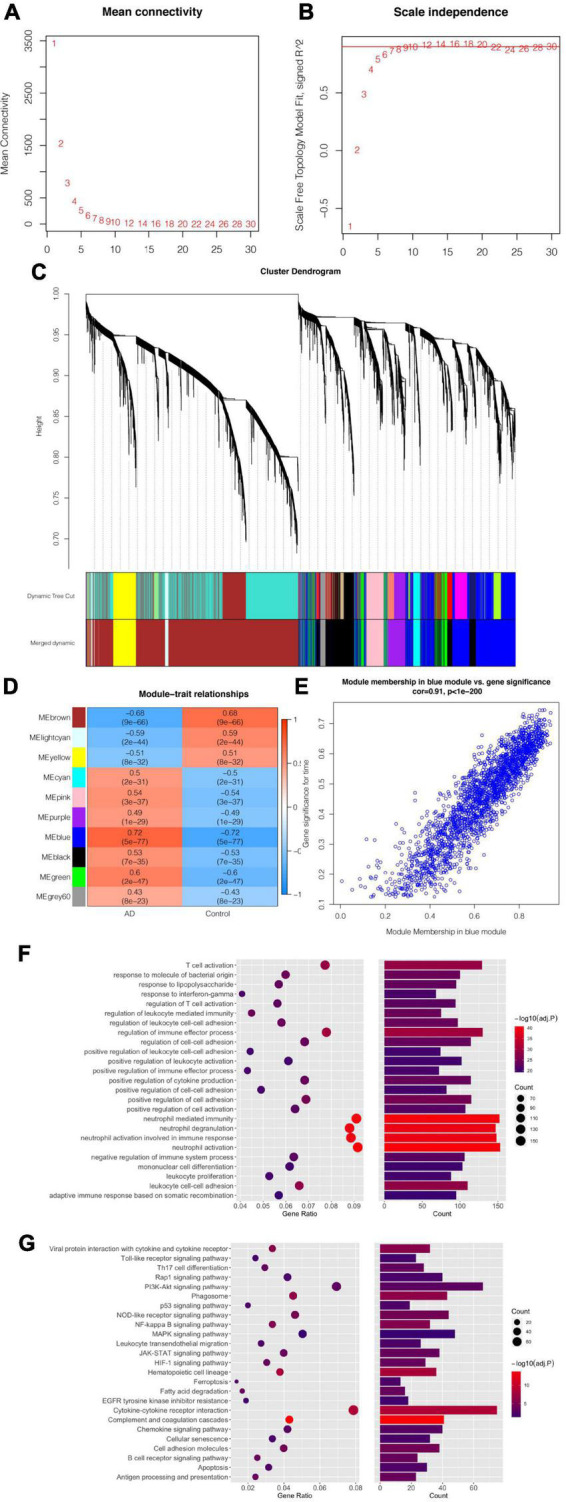
Weighted correlation network analysis of DEGs and Enrichment analysis result for the identified module. **(A,B)** Analysis of the scale independence and the mean connectivity for various soft-threshold powers. **(C)** The cluster dendrogram of 10,044 DEGs, with 10 modules in different colors. **(D)** Heatmap of the correlation between modules and phenotypes. Red shows a positive correlation and blue shows a negative correlation. Each cell contains the correlation coefficients. **(E)** A scatterplot of Gene Significance (GS) vs. Module Membership (MM) in the blue. Hub genes are evaluated by GS > 0.7 and MM > 0.8. **(F)** Gene Ontology enrichment analysis. **(G)** Kyoto Encyclopedia of Genes and Genomes enrichment analysis.

Through the application of WGCNA analysis, the expression values of 10,044 genes were used to construct a weighted co-expression network. In order to construct a scale-free network simulating a true biological network, the power value was selected to be 8 and the independence degree was ≥0.9 (Figures 4A,B). In this analysis, ten modules were detected according to similar expression characteristics (Figure 4C). Cluster analysis showed that various modules were related to AD, however, we need to identified the co-expression module most relevant to clinical features. Among the extensive number of modules, the blue module was analyzed further as it exhibited a highly correlation with AD (correlation coefficient = 0.72, *P* = 5E-77; Figure 4D). on the basis of GS > 0.7 and MM > 0.8 (Figure 4E), we identified 125 hub genes shared by the blue module.

### Functional and pathway enrichment analysis

Subsequently, to discover the potential molecular biological process, we conducted GO and KEGG enrichment analysis in the blue gene clusters. GO terms of molecular function revealed that these genes (Figure 4F) are primarily involved in regulation of immune cell activation and migration, cell-cell adhesion. The results of the KEGG pathway analysis indicated that inflammatory responses may be a key regulatory pathway associated with ferroptosis in AD, such as Immune cell differentiation, phagocytosis, cytokine interaction, antigen processing and presentation. Meanwhile, other regulatory pathways also included metabolic process (fatty acid metabolism), programmed cell death (apoptosis, ferroptosis), protein interaction, Toll-like receptors, and PI3K-Akt signaling pathway. Of these pathways, cell death and Toll-like receptors were closely associated with the role of ferroptosis in AD (Figure 4G).

### Identification and validation of hub genes related Alzheimer’s disease classifier

**FIGURE 5:**
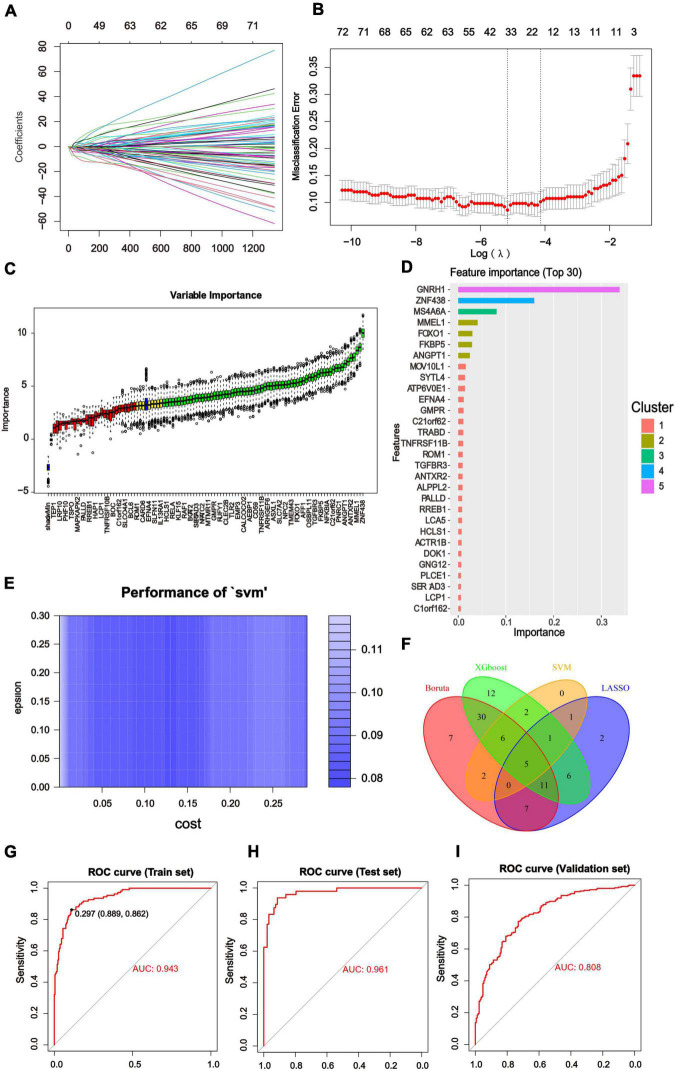
Results of Machine Learning Algorithms and ROC curve. **(A)** LASSO coefficient profiles of the significant hub genes. **(B)** Cross validation for turning parameter (lambda) selection in the LASSO regression. The LASSO model selected the log(λ) value for further analysis. **(C)** Screening conditions of Boruta algorithm. The green boxes confirmed the top 68 important features. The other two colors represented tentative and rejected attributes. **(D)** 125 hub genes identified by WGCNA were calculated by XGBoost and automatically ranked in order of importance. A list of the top 30 genes was predicted by XGboost classifier. **(E)** Using SVM modeling, 17 feature genes were extracted as gene biomarkers of AD from the aforementioned 125 hub genes. **(F)** Venn diagram to screen 5 overlapping genes presented in four Machine Learning Algorithms. **(G–I)** ROC curve was used to investigate the diagnostic model based on five diagnostic markers. The diagnostic model had the AUC value of 0.943 in the training set. The AUC of the test set was 0.961 and that of the validation set was 0.808. The *X*-axis represented the (1-specificity), and the *Y*-axis represented the sensitivity in the ROC curve.

We constructed diagnostic classifiers with four distinct algorithm types (LASSO, random forest, XGBoost and SVW). A feature selection strategy was performed to reduce the number of hub genes. The LASSO Cox regression model was employed to identify the most significant genes from 104 hub genes associated with AD (Figures 5A,B). We utilized Boruta algorithm to filtrate irrelevant features. The results indicated that it revealed 68 variables as the core genes (Figure 5C). The XGBoost model examined the importance of features and the top 30 indicators were displayed (Figure 5D). Similarly, using the SVM algorithm, we also obtained Feature screening of hub genes (Figure 5E). The hub genes obtained in the four algorithms were intersected by a Venn diagram accordingly, and 5 features were selected (RAF1, NFKBIA, MOV10L1, IQGAP1, FOXO1; Figure 5F).

### Establishment of Alzheimer’s disease predictive model

We divided the GSE33000 into training dataset (70%) and test dataset (30%) randomly and added the GSE5281/GSE48350 datasets for external validation. Then the above core genes underwent multivariable logistic regression analysis, identifying with non-zero regression coefficients. The optimized diagnostic model was calculated by the summation of “ExpRAF1×4.9453963+ExpNFKBIA×3.3202819 + ExpMOV10L1×7.7251910+ExpIQGAP1×0.8511138 + Exp FOXO1×1.3755429.” ROC analysis discriminated whether the 5-gene-based model had good diagnostic ability of AD. For instance, the area under the curve (AUC) was 0.943 in the training set, 0.961 in the test set and 0.808 in the validation set (Figures 5G–I). All of results indicated that the model had high predictive value in AD compared with normal samples and deserved further investigation.

## Discussion

With aging of the population in worldwide, AD is becoming a common issue. scientists are researching on diagnosis of AD in the earliest stages, before the toxic proteins damage large amounts of brain cells. However, as pathophysiological process precedes clinical symptoms (Sperling et al., 2011), AD is still poorly cured despite the availability of numerous clinical diagnostic methods. Therefore, if we can identify the factors related to the early onset of AD, it will be more conducive to the clinical diagnosis and treatment of disease. While dementia mechanism has developed rapidly over the past decade, dementia prediction models were analyzed to increase the diagnostic efficiency (Hou et al., 2019). We learned that multiple iron-regulatory proteins are abnormally expressed, leading to iron overload and accelerating the progression of AD (Wang et al., 2022a). In this study, we linked the diagnosis of AD with FRGs and used analyses to illustrate this correlation.

We performed a comprehensive bioinformatics analysis of hub FRGs involved in the pathogenesis of dementia, providing insights into the diagnosis. Firstly, we evaluated the expression level of 60 FRGs between AD and normal tissue based on GSE33000, and the screened genes were validated by GSE5281 and GSE48350 dataset. The results indicated that 8 genes were abnormally expressed in different region of the AD brain (AKR1C3, CD44, CISD1, CRYAB, GOT1, HMGCR, MT1G, NFE2L2). Among these genes, CD44 is identified as a potential biomarker for brain aging (Xu et al., 2022). As an inflammation-related gene, the increased expression of CD44 can promote the pathological progress of AD (Pinner et al., 2017). HMG-CoA reductase (HMGCR) is a rate-limiting enzyme involved in cholesterol synthesis (Zhang and Liu, 2015; Howe et al., 2017). Aβ can dramatically elevate the protein level of HMGCR, which may increase cholesterol synthesis (Cheraghzadeh et al., 2021). On the other, increasing of brain cholesterol level can exacerbate Aβ-induced neurotoxicity in AD (Fernandez et al., 2009; Li et al., 2018). The gene NFE2L2 encodes Nrf2, which is widely accepted to reduce oxidative stress and inflammation. Under oxidative stress induced by ferroptosis, free NRF2 is released and rapidly transferred to the nucleus, upregulating nuclear NRF2 (Alam et al., 1999; Sun R. et al., 2022). It further confirmed the association between FRGs and AD, providing more evidence for our study. Meanwhile, investigation of the remaining genes added novelty and innovation for subsequent molecular biology research. The above genes lay the foundation for discovery of diagnostic genes.

In this study we explored the specific regulation of FRGs on AD. Differential analysis was performed on the clustered dataset and GSE33000 to identify the DEGs, and the common DEGs were selected as candidate genes. That DEGs were mainly involved in the pathway of AD, neurotransmitter transmission, metabolic process, development of nervous system, and so on, which suggested regulatory factors associated with ferroptosis in AD. According to analyzing the expression patterns of candidate genes, a total of ten modules were proposed. After selecting the key modules, 104 hub genes were identified. In order to discover important pathways in biological processes, we did an enrichment analysis of hub genes, thus revealing the basic molecular mechanisms of biological processes.

Finally, machine learning algorithms were used to screen 5 potentially most relevant ferroptosis-related gene features (RAF1, NFKBIA, MOV10L1, IQGAP1, FOXO1), which were constructed a disease diagnostic model. All the five genes have been reported to be associated with neurodegenerative diseases, and FAF1, NFKBIA, FOXO1 are related to iron metabolism. The model can accurately classify patients from healthy individuals, indicating its potential value in molecular diagnosis.

RAF1 encodes protein named MAP kinase kinase kinase (MAP3K), playing an intermediate regulatory role in the linear RAS/RAF/MEK/ERK pathway (Ghousein et al., 2020). It was reported that RAF1 was involved in promoting neuronal neurite growth (Su et al., 2020). Moreover, RAF1 activation mediates cell death and survival, oncogenic transformation and hematopoietic function. Importantly, the heavy subunit of ferritin, FHC, can affect the gene expression of RAF1 (Pearson et al., 2001; Biamonte et al., 2015).

As for NFKBIA, it can encode IκBα to inhibit the function of NFκB. NFκB takes part in the inflammatory responses, anti-apoptotic transcription and angiogenesis regulation (Perkins, 1997). NF-κB activation is associated with neurodegeneration in AD, so NFKBIA is a candidate longevity−associated variant (Granic et al., 2009; Ryu et al., 2021). NFKBIA also have a strong ability of stabilizing mitochondria membrane (Pazarentzos, 2021). Iron-mediated cytotoxicity resulted in apoptosis accompanied by down-regulation of IκBα and up- regulation of NF-κB phosphorylation (Bhattacharyya et al., 2013).

MOV10L1 encodes an ATP-dependent RNA helicase (Wang et al., 2001) required for germline integrity, which is specifically expressed in germ cells. MOV10L1 regulates primary piRNA biogenesis and represses retrotransposons by forming complexes composed of piRNAs and Piwi proteins (Guan et al., 2021; Loubalova et al., 2021). For another, its paralog MOV10 is essential for normal brain circuitry and CNS function (Skariah et al., 2017). MOV10L1 might be due to Neuron development, for which further research is needed.

IQGAP1 participates various cellular functions, such as adherens junctions, cell migration, and cell proliferation. As a signal scaffolding protein, IQGAP1 regulates cell signaling transductions, such as MAPK signaling, Wnt Signaling, PI3K/Akt Signaling and TGF-β Signaling (White et al., 2012; Wei and Lambert, 2021). Many studies have reported that the overexpression of IQGAP1 contributes to different kinds of carcinoma (Takemoto et al., 2001; Zoheir et al., 2016; Wei and Lambert, 2021; Zhang Z. et al., 2022). It is also involved in the maintenance of neuronal function. IQGAP1 is identified as a key node of synaptic plasticity and dendritic spine density (Gao et al., 2011).

FOXO1 primarily regulates redox balance and osteoblast proliferation. Normal protein synthesis is necessary for redox balance. The interaction of FOXO1 and ATF4 maintain amino acid import and protein synthesis, which controlling osteoblast proliferation (Rached et al., 2010). In the nervous system, FOXO1 can affect neuronal autophagy (Castillo et al., 2013). On the other hand, FoxO1 is the main target of insulin signaling pathways, and as a result controls glucose metabolism (Kousteni, 2011; Nathanael et al., 2022). These mechanisms suggest FOXO1 may have implications in progression of AD. It is known that FOXO1-regulated HO1 overexpression increased the generation of ferrous iron (Dahyaleh et al., 2021). Ferroptosis may be related to the disease factor.

Currently, a few problems still existing in the diagnosis of AD. The pathological change of AD appears earlier than the symptoms. Upon onset of symptoms, diagnosis time can delay postpone treatment. There is increasing evidence of the importance of genetic factors in disease diagnosis. Several studies have used gene expression datasets downloaded from databases to clarify the biological mechanisms underlying AD development for disease prediction (Bellenguez et al., 2022; Wang et al., 2022b). On the basis of some research, ferroptosis has been shown to be involved in the pathological process of AD (Ayton et al., 2020; Sun Y. et al., 2022; Wang et al., 2022c). In the previous studies, to promote the development of diagnosis, researchers have explored candidate factors such as immune-based biomarkers (He et al., 2022), DNA methylation-related biomarkers (Chen et al., 2022) and aging-related biomarkers (Zhang Q. et al., 2022). The diagnostic link between ferroptosis and AD is not well studied. Consequently, we identified AD-related ferroptosis genes as candidate diagnostic biomarkers for AD and aimed to fill the gap by conducting bioinformatics analysis. Compared to similar previous studies, we applied conventional logistic regression and four different machine algorithms to validate diagnosis models creatively. The combination of the two methods is an excellent attempt to the existing diagnosis methods. Additionally, selected model genes have certain diagnostic value for clinical treatment. This conclusion also has profound significance for the scientific experimental study of ferroptosis. Results of genetic indicators and regulatory mechanism still need to be further tested by experiment. However, the verification measures have limitations as the difficulty of obtaining human brain samples. Moreover, there are high requirements for data analysis by lack of large datasets on AD. Further research requires more comprehensive genomic information and more normative clinical information.

In conclusion, we proposed five characteristic genes related to ferroptosis (RAF1, NFKBIA, MOV10L1, IQGAP1, FOXO1) in the diagnose of AD. In addition, further in other studies are required to verify the evidence for ferroptosis in the prevention and treatment in AD.

## Data availability statement

The original contributions presented in this study are included in the article/Supplementary material, further inquiries can be directed to the corresponding author.

## Author contributions

DH conceived and designed the study. YD and YF performed data analysis and wrote the manuscript. ZL collected data from database. JH, XC, and CW validated the analysis. MY and TX made the major effort of polishing the language. WG revised the manuscript. DC and HZ supervised the whole research. All authors contributed to the article and approved the submitted version.

## Footnotes

1
https://www.ncbi.nlm.nih.gov/geo/query/acc.cgi

2
https://www.gsea-msigdb.org/gsea/msigdb/index.jsp

3
https://www.gsea-msigdb.org/gsea/index.jsp
